# Risk implications of long-term global climate goals: overall conclusions of the ICA-RUS project

**DOI:** 10.1007/s11625-018-0530-0

**Published:** 2018-01-29

**Authors:** Seita Emori, Kiyoshi Takahashi, Yoshiki Yamagata, Shinjiro Kanae, Shunsuke Mori, Yuko Fujigaki

**Affiliations:** 1 National Institute for Environmental Studies, 16-2 Onogawa, Tsukuba, Ibaraki Japan; 2 Tokyo Institute of Technology, 2-2-1 Ookayama, Meguro, Tokyo Japan; 3 Tokyo University of Science, 2641 Yamazaki, Noda, Chiba Japan; 4 The University of Tokyo, 3-8-1 Komaba, Meguro, Tokyo Japan

**Keywords:** Climate change risks, Impact assessment, Integrated assessment, Paris Agreement, Social aspects of risk decisions

## Abstract

We have assessed the risks associated with setting 1.5, 2.0, or 2.5 °C temperature goals and ways to manage them in a systematic manner and discussed their implications. The results suggest that, given the uncertainties in climate sensitivity, “net zero emissions of anthropogenic greenhouse gases in the second half of this century” is a more actionable goal for society than the 2 or 1.5 °C temperature goals themselves. If the climate sensitivity is proven to be relatively high and the temperature goals are not met even when the net zero emission goal is achieved, the options left are: (A) accepting/adapting to a warmer world, (B) boosting mitigation, and (C) climate geoengineering, or any combination of these. This decision should be made based on a deeper discussion of risks associated with each option. We also suggest the need to consider a wider range of policies: not only climate policies, but also broader “sustainability policies”, and to envisage more innovative solutions than what integrated assessment models can currently illustrate. Finally, based on a consideration of social aspects of risk decisions, we recommend the establishment of a panel of “intermediate layer” experts, who support decision-making by citizens as well as social and ethical thinking by policy makers.

## Introduction

Climate change caused by human activities mainly through increasing concentrations of atmospheric greenhouse gases (GHGs) is one of the major threats to the current civilization of humankind. Since the United Nations Framework Convention on Climate Change (UNFCCC) aiming at “preventing dangerous anthropogenic interference with the climate system” was established in 1992 (United Nations 1992), long-term goals of climate change mitigation in more concrete terms have long been discussed both scientifically and politically (Randalls 2010; UNFCCC 2015a). As a culmination of such discussions, a set of statements on long-term climate goals were included in the Paris Agreement which was agreed by the international community under UNFCCC in 2015 and came into force in the following year (UNFCCC 2015b). Namely, “holding the increase in the global average temperature to well below 2 °C above pre-industrial levels and pursuing efforts to limit the temperature increase to 1.5 °C above pre-industrial levels” was agreed (hereafter, referred to as “the Paris temperature goals”). To be roughly consistent with this, to “achieve a balance between anthropogenic emissions by sources and removals by sinks of greenhouse gases in the second half of this century” is also stated in the agreement (“the Paris emission goal”), which essentially means reducing net global anthropogenic GHG emissions to zero sometime between 2051 and 2100.

It has been widely recognized that such a goal cannot be determined based solely on science, as it involves decisions under uncertainties and diverse value judgments (O’Neill et al. 2017). Nonetheless, obviously, it should be underpinned by science. Tremendous efforts have been made so far to assess risks due to climate change impacts for different temperature levels. To be as comprehensive as possible, some efforts rely on extensive surveys of assessments in the existing literature (IPCC 2014a; Warren et al. 2015), which are usually based on diverse conditions, assumptions, and scenarios. There have been some attempts of multiple-sector impact assessments based on consistent sets of underlying scenarios (Arnell et al. 2014; Schleussner et al. 2016a), but that kind of work heavily depends on coordination efforts and thus are still very limited. Mitigation scenarios have also been extensively assessed through literature surveys or a dedicated database as a community effort (IPCC 2014b; Schleussner et al. 2016b). Attempts to combine assessments of impacts and response options are still rare except for the Synthesis Reports of IPCC (IPCC 2014c).

The ICA-RUS project (“Integrated Climate Assessment—Risks, Uncertainties and Society”) was commenced in 2012 as a “Comprehensive Research on the Development of Global Climate Change Risk Management Strategies” S-10 Strategic Research Project supported by the Environmental Research and Technology Fund of the Ministry of the Environment of Japan, and finished its 5-year plan in March, 2017. It was an inter-disciplinary research project pulling together experts of climate science, impact assessment, energy economics, and studies on science and technology from various institutes and universities in Japan, aiming at assessing climate risks and ways to manage them in a systematic manner. Motivated partly by a reflection on the Fukushima nuclear crisis that Japanese society had experienced in 2011, just before we started the project, we framed the climate issue as a risk management problem at a global scale. By the term risk management, we mean (1) decision-making under uncertainty, (2) decision-making based on scientific evidence, (3) consideration, insofar as possible, of all kinds of circumstances and options, (4) flexible revision according to changes in conditions, and (5) involvement of social value judgments. Viewpoints 3–5 were highlighted particularly in the wake of Fukushima, as we thought the Fukushima case seemed to lack an adequate consideration of these aspects of risk management (Fujigaki 2015). From a risk management perspective, incorporation of a full range of uncertainties into decision-making is required, which has not been successfully done in most of the discussions on climate goals so far (Mabey et al. 2011). Note that, although climate risk management often means climate change adaptation and disaster management at a local scale (e.g., Fünfgeld 2010), in the context of our “global climate risk management”, adaptation is not necessarily the main focus and is regarded only as one of the options.

Research in the ICA-RUS project was undertaken in the following five themes: (1) synthesis of global climate risk management strategies, (2) optimization of land, water, and ecosystem uses for climate risk management, (3) identification and analysis of critical climate risks, (4) evaluation of climate risk management options under technological, social, and economic uncertainties, and (5) interactions between scientific and social rationalities in climate risk management. This paper presents the overall conclusions from the discussion in ICA-RUS invoked by this inter-disciplinary research, following brief descriptions of the design and results of the essential parts of assessment done in the project. A fuller project report is available at the project website (http://www.nies.go.jp/ica-rus/en/materials.html).

## “Strategies” defined and assessed in ICA-RUS

**Fig. 1:**
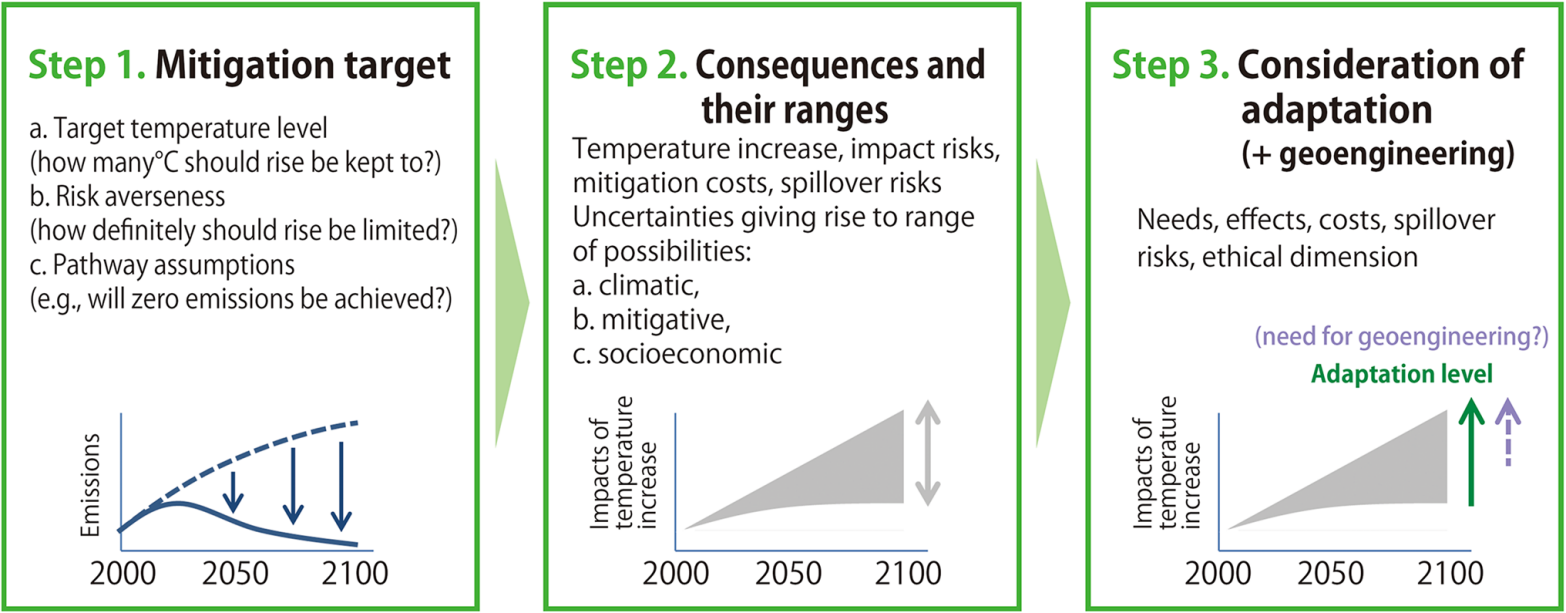
Concept of “strategy” used by ICA-RUS

In ICA-RUS, we developed a concept of “strategies” of global climate risk management and defined through three steps described below (Fig. 1). The “strategies” are treated as risk management alternatives that are available to society. Note that the subject adopting a “strategy” in this sense is intended to be the human society or the global community as a whole through collective decision-making and actions, rather than any particular actor or group of actors. Breaking down a “strategy” into decisions and actions of each actor needs further discussion, which is not covered in this study.

Step 1: Setting mitigation targets.

Mitigation targets are defined by three considerations: (a) target temperature level, (b) risk averseness, and (c) assumptions about the pathway. Here, the choice regarding risk averseness means deciding on the basis of scientific uncertainty how likely it is that the target temperature would be exceeded. The assumptions about the pathway include a choice as to whether “overshoot” (the concentration or temperature decreases after an initial increase) is allowed or not (Huntingford and Lowe 2007). The pathways of global GHG emissions consistent with these considerations were identified in Step 1.

Step 2: Deriving a range of consequences for each mitigation target under uncertainties.

The range of possible consequences for each mitigation target is determined taking into account various uncertainties, such as climate (and impact) uncertainties, uncertainties concerning mitigation actions, and socio-economic uncertainties. Climate uncertainties arise from uncertainties in the scientific estimates of factors such as climate sensitivity (Freeman et al. 2015). Uncertainties in mitigation actions arise from the possibility that a mitigation action considered necessary to achieve a target is only partially implemented, or else is fully implemented but does not prove as effective as anticipated, and gives rise to the risk that the temperature rise may be greater than initially projected (Mabey et al. 2011). Socio-economic uncertainties (excluding those relating to climate policy) arise from uncertainties in future projections of variables such as world population, economic development, and social inequality (Riahi et al. 2017).

Step 3: Considering necessary adaptation levels (plus need for geoengineering).

The necessary level of adaptation action is considered for each mitigation target and costs are estimated where possible. As a range of consequences is possible for each mitigation target due to the existence of various uncertainties, there is a risk that the temperature rise may exceed the target. It is, therefore, important to take this possibility into account when considering what adaptation action will be required. Where a mitigation target remains subject to the risk of a large temperature rise, consideration should also be given to geoengineering, and in particular to the use of solar radiation management (Royal Society 2009).

In practice, different “strategies” based on the above concept were only partially explored in ICA-RUS. In Step 1, different levels of overshoot were not assessed. In Step 2, uncertainties only due to climate sensitivity and socio-economic scenarios were explicitly considered. Finally, Step 3 was not systematically assessed and was discussed only conceptually. In particular, the lack of a comprehensive assessment of adaptation is a notable limitation of the project. Although we did assess certain aspects of adaptation for agriculture and health, the results are not described in this paper as it focuses on the overall results. None the less, we believe that the concept is useful for further research in the future, where those uncertainties and factors are more fully assessed.

In ICA-RUS, we explored several “strategies” with different combinations of target temperature level and risk averseness. In this paper, however, we mainly discuss only three “strategies” which we eventually focus on. They are T15, T20, and T25 with the GHG emission scenario that would have a 66% probability of limiting the global mean temperature rise to no more than 1.5, 2.0, and 2.5 °C, respectively, above the pre-industrial levels. From the next section, they are simply referred to as three different temperature goals rather than “strategies”.

To briefly summarize our findings on different risk averseness, the results for a “strategy” with a 2.0 °C limit and an 80% probability for staying below the limit were broadly similar to those for 1.5 °C and 50%. Likewise, a “strategy” with a 2.5 °C limit and an 80% probability produced similar results for 2.0 °C and 50%. Although a 66% probability was adopted for the main “strategies” in ICA-RUS, as it is frequently used in the literature (e.g., Rogelj et al. 2015), we argue that the choice of risk averseness is one of the central factors in risk management and needs more discussion.

## Assessment of impacts of climate change and mitigation actions for each temperature goal

To identify emission pathways in Step 1 that were consistent with the stated mitigation targets, we developed a dynamic optimization model SCM4OPT (Su et al. 2017) based on the Dynamic Integrated Climate-Economy (DICE) model (Nordhaus 1992). To be able to apply the model for the analyses of ambitious targets such as a temperature increase of no more than 1.5 °C, it was designed to explicitly consider in its economic module mitigation of emissions of CO_2_ as well as other gases/aerosols. The simple climate module in the DICE model was also improved to treat the behavior of the other gases/aerosols individually. See also Su et al. (2018) in this Special Feature.

For the assessment of the impacts of climate change corresponding to each temperature goal, process-based impact models for multiple sectors were used for future projections of impacts. The sectors covered are (a) agriculture (Sakurai et al. 2014), (b) terrestrial ecosystems (Ito et al. 2016), (c) water resources (Hanasaki et al. 2013), (d) floods (Hirabayashi et al. 2013), (e) health (Honda et al. 2014), and (f) marine bio-geochemical cycles and ecosystems (Yamamoto et al. 2014) (17 variables in total). The impact model analyses was coordinated to use the same set of climate scenarios from the selected five climate models which participated in the fifth phase of the Coupled Model Intercomparison Project (CMIP5; Taylor et al. 2012), namely GFDL-ESM2M, HadGEM2-ES, IPSL-CM5A-LR, MIROCESM-CHEM, and NorESM1-M, for four levels (2.6, 4.5, 6.0, and 8.5) of Representative Concentration Pathways (RCP; Moss et al. 2010) and three Shared Socio-economic Pathways (SSP1, 2 and 3; Riahi et al. 2017). The results of the impact models for the RCP scenarios were linearly interpolated according to global mean temperatures to estimate the impacts for each temperature goal. The validity of this linear scaling is discussed by Tanaka et al. (2017).

The five climate models selected to represent climate uncertainties largely cover the upper and lower limits of the ranges of global mean temperature and precipitation projections generated by the CMIP5 climate model ensemble. However, they should be regarded as being only for illustrative purposes for detailed aspects of the projections such as geographical distributions and characteristics of temporal variabilities. As only a single impact model was used for each sector, uncertainties in the impact models were not explored. The socio-economic pathways were selected to explore a range of different levels of sustainability. Namely, in our project, the SSP1 was the most sustainable, followed by SSP2, and SSP3 was the least sustainable pathway, judged from their narratives and baseline GHG emissions, among other indicators.

For the assessment of portfolios of mitigation options and associated economic impacts to achieve an emission pathway for each temperature goal, multiple integrated assessment models (IAMs), namely, MARIA (Mori 2000), AIM (Fujimori et al. 2012), EMEDA (Washida et al. 2014), and GRAPE (Kurosawa 2006), were employed. The use of multiple models enabled us to illustrate an indication of uncertainties caused by different model structures and assumptions. The variables assessed were GHG emissions and reduction pathways, GDP and consumption loss, energy supply and demand, technological options, land use and food supply and demand, and impacts for each industry and region. See Mori et al. (2018) in this Special Feature for more details of this part of ICA-RUS.

## Overall results of the assessment

**Fig. 2:**
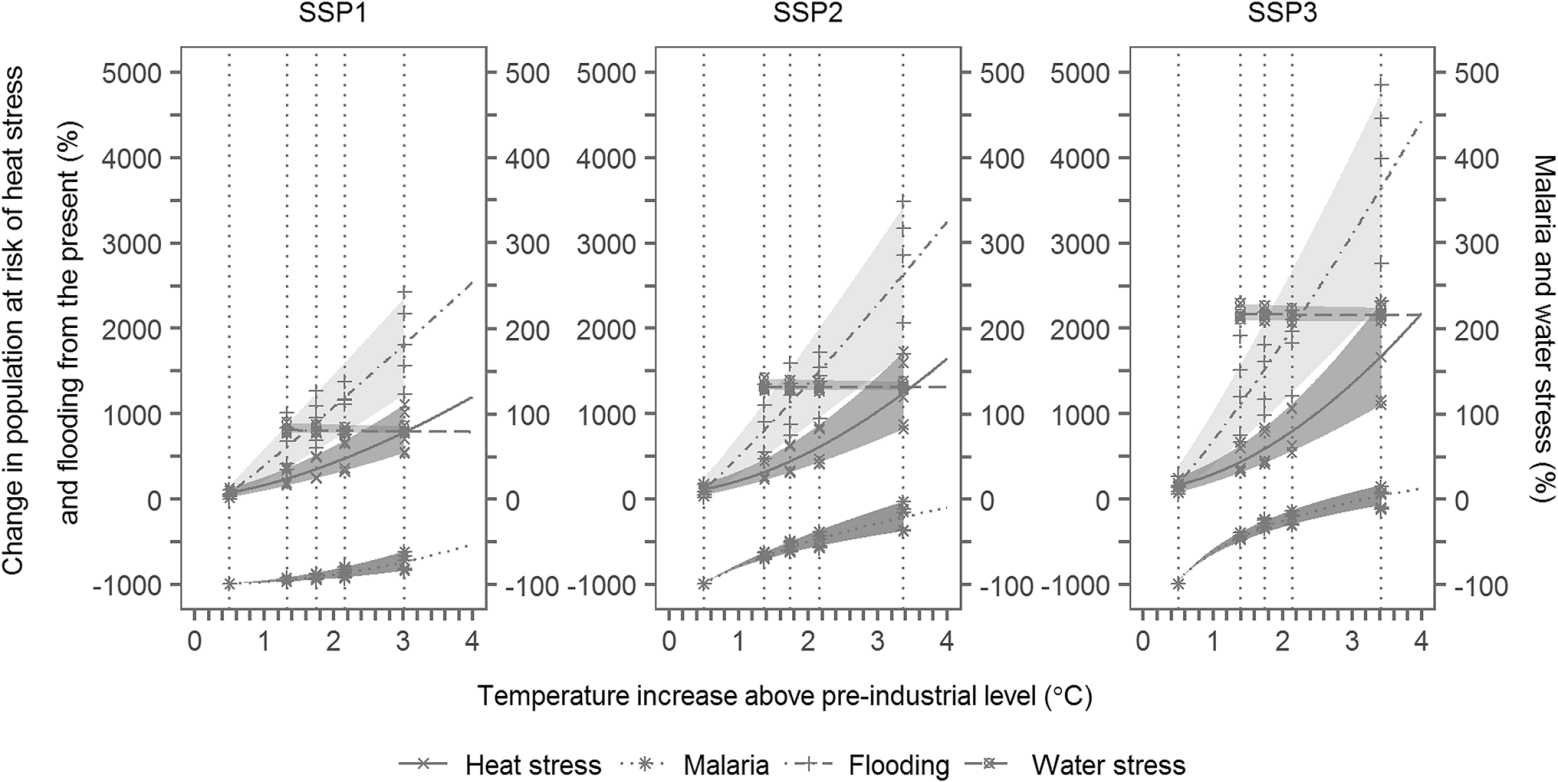
Impacts of climate change in the 2080s in terms of relative change in global affected populations for selected sectors (heat stress, malaria, flooding, and water stress) depending on different goals (T15, T20, and T25) as well as BaU and fixed climate cases, for three different socio-economic pathways (SSP1-3). The abscissa is a global mean temperature change in the 2080s relative to pre-industrial levels for each case, which are 0.5, 1.4, 1.8, 2.2, and 3.0 °C (or over, depending on SSP) for the fixed climate case, T15, T20, T25, and BaU, respectively. Shades represent the uncertainty range assessed using multiple climate models. The water-stress impact for the fixed climate case is not shown as it was not calculated

Figure 2 summarizes our climate change impact assessment at a global scale. Though our analysis also provides regional information, we focus on the overall results for brevity in this paper, keeping in mind that regional impacts could be essential in the assessment of different goals. The impacts in terms of relative change in affected populations for selected sectors are shown depending on different goals (T15, T20, and T25) as well as business as usual (BaU; without mitigation actions) and fixed climate cases, for three different socio-economic pathways (SSP1-3). The fixed climate case shown at 0.5 °C temperature increase from the pre-industrial level represents an imaginary case only with socio-economic development occurring without climate change, as a reference. Climate scenarios from multiple climate models were used to represent uncertainty ranges. The populations affected by heat stress (defined by the number of additional heat-related deaths) and flooding (the population living in inundated areas) increase from the current values with increasing temperature. The malaria-affected population (the population living in malaria endemic areas) would be reduced to close to zero due to socio-economic development in the fixed climate case, but the reduction would be less for a larger temperature increase. The water-stressed population (the population living in areas where per capita runoff is less than 1700 m^3^/capita/year, based on Falkenmark 1989) shows a slight decrease with increasing temperature due to increasing precipitation. The affected populations are larger for a less sustainable socio-economic pathway mainly due to a greater population increase.

**Fig. 3:**
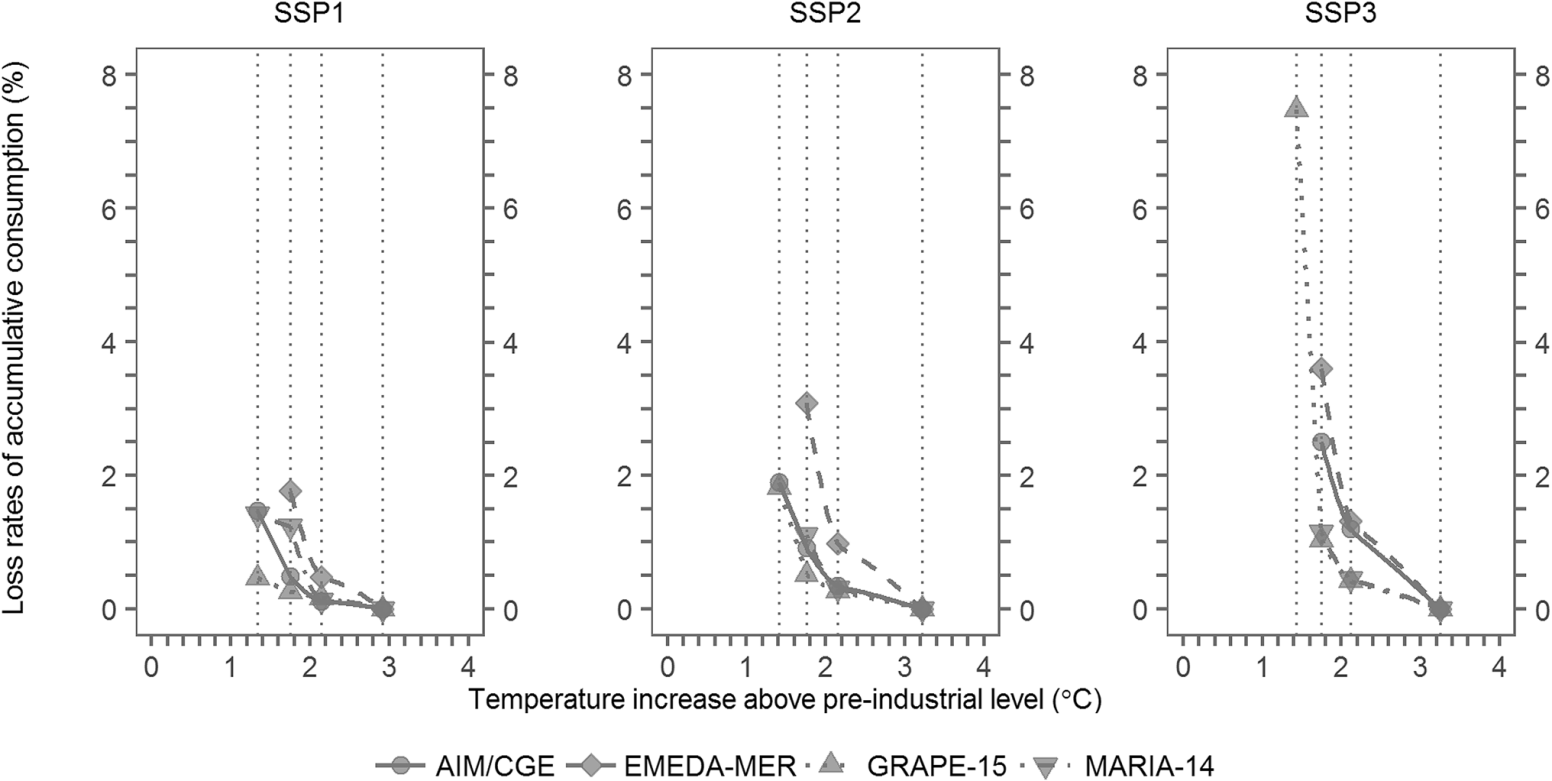
Loss rates of global cumulative consumption (from present to the 2080s) for different goals (T15, T20, and T25) and BaU and for three different socio-economic pathways (SSP1-3). The abscissa is a global mean temperature change in the 2080s relative to pre-industrial levels for each case, which are 1.4, 1.8, 2.2, and 3.0 °C (or over, depending on SSP) for T15, T20, T25, and BaU, respectively. The results from four integrated assessment models are shown

Figure 3 shows the cumulative consumption loss for different goals (including BaU) and socio-economic pathways, as a rough indicator of the magnitude of mitigation efforts needed for each case, derived from our assessment of economic impacts of mitigation actions. The results from four IAMs are shown to represent an indication of uncertainty. A discount rate of 5% was used for calculating the present values before temporal accumulation, but a different discount rate (ranging from 0 to 5%) changes only the overall scale and not the shapes of the graphs (not shown). The cumulative consumption loss naturally increases with decreasing temperature (i.e., increasing ambition of a goal). Note that, for the T15 goal, some of the IAMs used in ICA-RUS could not provide a feasible solution. For SSP1, only one model was unable to solve the T15 case. For SSP2 and 3, the number of models unable to solve the T15 case increased to two and three, respectively. This clearly demonstrates that the T15 goal is less feasible for a less sustainable socio-economic pathway.

Comparing the two figures, we can see that the climate change impacts are generally less sensitive to the choice of a goal than to the magnitude of efforts needed to achieve a certain goal. The impacts for any goal (T15, T20, or T25) are substantially smaller than those for BaU (except for water stress, for which the impact is insensitive to mitigation actions). However, the difference in impacts between any two different goals is generally smaller than or comparable to climate uncertainties. On the other hand, mitigation efforts increase sharply with increasing ambition of a goal, and some models cannot provide a feasible solution when a goal is more ambitious than a certain limit, although the inter-model spread is considerably large.

We do not claim any quantitative (cost–benefit type) conclusion from this comparison, as all the impacts cannot be represented in economic terms and the sectors covered are limited. Moreover, there are many unresolved limitations and disputed assumptions for a globally aggregated economic assessment of climate change in general (IPCC 2014c). Having said that, the existing estimates available so far with known limitations do not necessarily show that economic loss due to climate change impacts is greater than that due to mitigation actions. For example, according to IPCC (2014c), global annual economic losses for warming of ~ 2.5 °C above pre-industrial levels are 0.2–2.0% of income, while global consumption losses for mitigation scenarios to limit warming to below 2 °C through the 21st century relative to pre-industrial levels are 3–11% in 2100 relative to baseline scenarios, though the numbers are not directly comparable. With this rough quantitative consideration in mind, we can suggest at least that a preference to a more ambitious goal (e.g., T15 as compared to T20) is not necessarily supported by our globally aggregated assessment. Rather, seen from a risk management perspective, we argue that how climate uncertainties (e.g., risks due to high climate sensitivity) and risks of failure to achieve a goal are managed are as important as the choice of a goal.

## Discussion

### Interpretation of the Paris goals

In this section, we present an interpretation of the Paris goals in the light of our assessment described above (The ICA-RUS project was originally intended to provide alternatives in terms of long-term climate goals. However, in the Paris Agreement, the international community has already chosen long-term goals. Therefore, we changed the purpose of our project and made an interpretation of the Paris goals.)

First, we found in the above that temperature goals as low as 2 or 1.5 °C cannot necessarily be supported from a globally aggregated cost–benefit perspective, as far as we can tell within the scope of our assessment. Although maximizing the aggregated economic value is often seen as an objective principle to optimize a policy intervention, we should note that the adoption of this principle, which is a form of utilitarian approach, heavily involves value judgments. By value judgments, we mean judgments on a question like “what should be protected?”, “what should be avoided?”, or “what is acceptable/unacceptable?” To appreciate the grounds of the Paris temperature goals, values other than the globally aggregated economic value should be taken into consideration. As has been discussed in the literature (Okereke 2010), such values involve ethical dimensions, particularly a concept called “climate justice”, which draws attention to a structural social injustice that those who are least responsible (i.e., people in developing countries and future generations, who have emitted least amount of GHGs) suffer the most harm. The Paris temperature goals can be supported by an ethical position that such an inequality is unacceptable. Other grounds may come from concerns of crossing a “tipping point” of any sub-system of the Earth system (tipping element). Although the scientific understanding on tipping elements still has large uncertainties, and thus, our assessment of them is also limited; the lowest possible temperature goals might well be supported to reduce the risks of crossing any tipping point. This could also involve value judgments, as triggering a catastrophic change in the Earth system by the current generation, which entails grave consequences for future generations, is unacceptable by a certain ethical position. The issue of tipping elements is further discussed by Iseri et al. (2018) in this Special Feature.

Second, actions needed to meet a temperature goal are not very clear, given the uncertainty in climate system (including climate sensitivity, carbon-cycle feedback, and possibly others). On the other hand, the emission goal of the Paris Agreement (net zero global anthropogenic GHG emissions in the second half of this century) is a clearer one in terms of actions needed. In that sense, seen from the framework of our assessment, we appreciate the Paris emission goal as a more “actionable” goal. The two kinds of goals, the temperature and emission goals, are roughly, but not precisely, consistent with each other, and the relationship of the two should be made clearer. This conclusion of our echoes that of Geden (2016)’s, who also recommended a net zero emissions’ target as an actionable one, although his conclusion was based on his political science arguments.

### Learning climate sensitivity

Practically, we should start taking actions with estimated likely values (or ranges) of climate parameters and then adjust our mitigation pathways in the course according to the future reductions of uncertainties in climate parameters (so-called “learning” or “act then learn” approach; Yohe et al. 2004; Manne and Richels 1992). Hereafter, for simplicity, we use “climate sensitivity” as a single parameter representing uncertain processes in the Earth system, keeping in mind that other parameters (including the one for carbon-cycle feedback) can be important, as well (Friedlingstein et al. 2014).

The estimate of climate sensitivity is expected to be improved through an advancement of science. However, even without such an advancement, thanks to the future accumulation of the observed surface air temperature data, the uncertainty range of global mean temperature changes can be reduced (Shiogama et al. 2016). We suggest that this gradual improvement of the estimate of climate sensitivity through learning plays an essential role when we deal with uncertainties lying between the temperature and emission goals. The economic value of scientific information in this context is discussed by Mori and Shiogama (2018) in this Special Feature.

If we find in this process that the climate sensitivity is substantially lower than the current central estimate [~ 3 °C, the middle of likely range from IPCC (2013)], we can more reasonably aim at the 1.5 °C goal, as the emission path for aiming at 1.5 °C goal with climate sensitivity of a little more than 2 °C is almost equivalent to that for the 2 °C goal with a climate sensitivity of 3 °C.

On the contrary, in case we find that the climate sensitivity is substantially higher than the central estimate, we should recognize that we are on the course of exceeding 2 °C even when the emission goal is achieved. This case represents a possible gap between the Paris temperature and emission goals and needs more discussion.

### Alternatives left for the case of high climate sensitivity

We have identified the following three options that we could take if we found that climate sensitivity was substantially high and 2 °C would be exceeded even if the Paris emission goal was achieved.

- Option A: Accepting and adapting to a warmer world
- Option B: Boosting mitigation
- Option C: Climate geoengineering.

Option A means taking no additional mitigation action and letting the temperature exceed 2 °C, accepting the risks of more severe climate change impacts, and possibly making efforts and paying costs for additional adaptation.

Option B means accelerating mitigation actions exceeding the Paris emission goal, which possibly means that we should aim at substantially net negative GHG emissions in the second half of this century, accepting additional costs and risks due to possible side-effects. For example, bio-energy with CCS (BECCS) is one of the negative emission technologies that could be adopted then (and is often adopted in many scenarios to achieve even the Paris emission goal), but its large-scale deployment requires huge land areas and might compromise food security and/or ecosystem services (Fuss et al. 2014; Kato and Yamagata 2014). See also Yamagata et al. (2018) in this Special Feature.

Option C basically refers to the so-called solar radiation management, which is to cancel the warming effect of increased GHGs by the reduction of incoming solar radiation due to stratospheric aerosol injection or other means (Royal Society 2009). The adoption of this option means to accept risks due to possible side-effects and governance failure associated with this techno-fix solution (Preston 2013).

Obviously, no option is easy to adopt, as any of them entails additional risks, which can be disastrous. However, the international community should prepare to choose one or any combination of them, in case we find that climate sensitivity is high. That decision will require the deepest understanding and most careful deliberation of scientific and moral aspects of risks associated with each option.

### Remarks on the achievement of the Paris emission goal

The above discussion is based on the assumption that we are able to achieve the Paris emission goal, that is, net zero global anthropogenic GHG emissions in the second half of this century. However, it is by no means an easy goal to achieve.

One remark regarding the achievement of the Paris emission goal that we can make based on our assessment is that the mitigation action needed to achieve the goal heavily depends on the socio-economic pathways, as has been observed in past assessments (IPCC 2014b), and is smaller for a more sustainable pathway, i.e., the smallest for SSP1, followed by SSP2, and the largest for SSP3, in our assessment. Different socio-economic pathways could be interpreted as representations of the uncertainty range of socio-economic development that we cannot manage. They are certainly external factors when we only think of “climate policy” in a narrow sense, which is represented in IAMs as introducing more expensive mitigation options by setting a higher carbon price. However, when we think of a wider “sustainability policy”, policy interventions should be able to affect SSPs to some extent. Although we did not clarify or identify within the scope of our project what such sustainability policies were, we can suggest that policies to shift the socio-economic condition of the world to a more sustainable one (e.g., from SSP2 toward SSP1) are as important as the climate policy itself to achieve the Paris emission goal, and they should be seriously discussed and implemented.

Another remark is about the limitation of scenarios illustrated by IAMs and the need for a careful interpretation of them. In principle, IAMs cannot represent structural changes (or “transformation”) of socio-economic and technological systems that cannot be foreseen at present, especially when they are applied to illustrate scenarios until the end of this century. Therefore, mitigation scenarios are not something that we have to follow exactly and expected economic damages are not necessarily what we have to accept. Instead, what really is necessary is creating policies to relax constraining conditions that are assumed in models and facilitating transformation of the systems. This point might be obvious in some research communities (Geels et al. 2016), but not necessarily in others. It was not drawn directly from our assessment, but was brought up in our inter-disciplinary discussion. At the same time, we should not forget that IAMs cannot represent either possible political failures that might result in insufficient implementation of mitigation actions (Mabey et al. 2011). Continuous efforts to avoid such a failure are needed.

### Social process of risk decision on global and long-term climate change

The decision to choose global and long-term goals or options as described in Sect. 5.3 cannot be made based solely on science. It involves uncertainties and value judgments. Thus, social processes play an essential role in such a decision-making, possibly involving citizen participation to democratize the process.

However, we found in our survey that climate change, as compared with other risk issues, has a characteristic that an average person typically feels that it is difficult to be engaged in, as it is complex, uncertain, long-term, and hard-to-feel self-efficacy (Moser 2010; Wolf and Moser 2011). Nonetheless, citizens do not necessarily want authorities to make the whole decision. There is a gap between the understanding of climate risk issues by experts and that by citizens. Experts’ understanding should be translated into contexts of citizens’ everyday lives. In addition, citizens may want to know the ethical dimensions and value judgments that are embedded in seemingly objective experts’ arguments.

To bridge this gap, we propose that an “intermediate layer” of experts be designed to mediate among scientific experts, citizens, and stakeholders to deal with ethical dimensions, value judgments, and translations. This layer will consist of experts from various disciplines particularly including the humanities and social sciences, who can translate scientific results into the contexts of everyday life and can explain how value judgements are embedded in certain scientific statements by revealing the hidden power and knowledge structures of society. The selection process of the members of this layer should be done by the government as well as by the public. The members of this layer should have an open meeting day with concerned people in public. The quality control of this layer will be ensured by continuous discussion. This proposal is discussed in detail by Fujigaki (2018) in this Special Feature.

## Conclusions

We have assessed the risks associated with setting 1.5, 2.0, or 2.5 °C temperature goals and ways to manage them in a systematic manner and discussed their implications. Process-based impact models for multiple sectors were used for future projections of impacts, while multiple IAMs were employed for assessing the portfolios of mitigation options and associated economic impacts for achieving an emission pathway for each goal.

The results suggest the following two major implications for ways to pursue the Paris Agreement goals. First, given the uncertainties in climate sensitivity, “net zero GHG emissions in the second half of this century” is a more actionable goal for society than the 2 or 1.5 °C temperature goals themselves. If the climate sensitivity was relatively high, the temperature goals would not be met even when the net zero emission goal was achieved. If it is proven (through “learning”) to be the case, the options left are: (A) accepting/adapting to a warmer world, (B) boosting mitigation, (C) climate geoengineering, or any combination of these. In this case, the decision should be made based on a deeper discussion of risks associated with each option in society with the latest knowledge on scientific and moral aspects of those risks.

Second, the net zero GHG emission itself is obviously not an easy goal to achieve. We need to consider a wide range of policies: not only climate policies (represented in IAMs by introducing more expensive mitigation options due to higher carbon pricing given a fixed socio-economic scenario), but also broader “sustainability policies” (policies to shift the socio-economic scenario toward a more sustainable one). We need to be aware that IAMs cannot represent innovative changes in technological and socio-economic systems that we are not able to foresee so far. The limitations for net zero emission scenarios (e.g., extremely high cost) would not be absolute. We can envisage more innovative solutions than what IAMs can currently illustrate. At the same time, we need continuous efforts to avoid any possible political failure, which is not represented either in IAMs.

Based on a consideration of social aspects of risk decisions, we also recommend the establishment of a panel of “intermediate layer” experts, who support decision-making by citizens as well as social and ethical thinking by policy makers. Although a global perspective was primarily adopted in our study, diverse subjects such as countries, local governments, enterprises, citizens, etc., need to advance the interpretation of climate risk implications in their own contexts. Conversely, opinions expressed based on their diverse contexts need to be delivered to the global review process. The intermediary experts are expected to facilitate this bi-directional process, but considering the implementation of this layer in society is a future task.
